# Radioiodine remnant ablation of differentiated thyroid cancer does not further increase oxidative damage to membrane lipids - early effect

**DOI:** 10.1186/1756-6614-3-7

**Published:** 2010-10-06

**Authors:** Jacek Makarewicz, Andrzej Lewiński, Małgorzata Karbownik-Lewińska

**Affiliations:** 1 Department of Nuclear Medicine and Oncological Endocrinology, District Hospital, Zgierz, Poland; 2 Chair and Department of Endocrinology and Metabolic Diseases, Medical University of Lodz, Poland; 3 Department of Oncological Endocrinology, Chair of Endocrinology and Metabolic Diseases, Medical University of Lodz, Poland; 4 Polish Mother's Memorial Hospital - Research Institute, Lodz, Poland

## Abstract

**Introduction:**

Radioiodine (^131^I) therapy is widely accepted as an essential part of therapeutic regimens in many cases of differentiated thyroid cancer. Radiation-induced oxidative damage to macromolecules is a well known phenomenon. Frequently examined process to evaluate oxidative damage to macromolecules is lipid peroxidation (LPO), resulting from oxidative damage to membrane lipids. The aim of the study was to examine serum LPO level in hypothyroid (after total thyroidectomy) cancer patients subjected to ablative activities of ^131^I.

**Materials and methods:**

The study was carried out in 21 patients (18 females and 3 males, average age 52.4 ± 16.5 years) after total thyroidectomy for papillary (17 patients) or follicular (4 patients) thyroid carcinoma. Hypothyroidism was confirmed by increased TSH blood concentration (BRAHMS, Germany), measured before ^131^I therapy. Activity of 2.8 - 6.9 GBq of ^131^I was administered to the patients orally as sodium iodide (OBRI, Poland). Concentrations of malondialdehyde + 4-hydroxyalkenals (MDA + 4-HDA), as an index of LPO (LPO-586 kit, Calbiochem, USA), were measured in blood serum just before ^131^I administration (day "0") and on the days 1-4 after ^131^I therapy. Sera from 23 euthyroid patients served as controls. Correlations between LPO and TSH or ^131^I activity were calculated.

**Results:**

Expectedly, serum LPO level, when measured before ^131^I therapy, was several times higher (p < 0.00001) in cancer patients than in healthy subjects, which is probably due to hypothyroidism caused by total thyroidectomy. However, we did not observe any differences between LPO levels after and before ^131^I therapy. LPO did not correlate with TSH concentration. In turn, negative correlation was found between ^131^I activity and LPO level on the day "2" after radioiodine treatment.

**Conclusions:**

Radioiodine remnant ablation of differentiated thyroid cancer does not further increase oxidative damage to membrane lipids, at least early, after therapy.

## Introduction

Radioiodine (^131^I) administration is a widely accepted method of therapy in both benign and malignant thyroid disorders. However, besides its desired effects on the thyroid tissue, ionizing radiation may cause cellular oxidative damage both directly, by disruption of DNA integrity, and indirectly, as a result of the formation of intracellular free radicals [1]. When the free radicals are generated closely to or within lipid membranes, they can attack the fatty acid side-chains of the membrane phospholipids and, then, lipid peroxidation (LPO) may occur [2]. Such a phenomenon relates practically to all cellular membranes of any tissue, and the products of LPO are thereafter translocated into body fluids, blood included.

Increased amounts of LPO end products can be, however, detected in many diseases, as an index of cell destruction, because cells and tissues damaged by any mechanism may peroxidize more rapidly than normal ones [3]. For example, increased LPO level was found in blood serum of critically ill patients, of adult growth hormone deficient patients, or of newborns with respiratory distress syndrome or with sepsis, as well as in rat blood serum after exposure to different carcinogens [4-9]. It should be stressed that the increased LPO was also described in blood serum of patients with hypothyroidism [10-13].

Well documented is radiation-induced oxidative damage to macromolecules [14]. Total body ionizing irradiation increased liver DNA and membrane lipid damage, as well as increased LPO level in blood serum in rats [15]. Also human tissues were documented to be sensitive to damaging effects of ionizing radiation. For example, exposure of human blood leukocytes to external irradiation increased DNA damage [16]. The hypothesis on the radiation-related processes of aging, via the mechanism of oxidative stress, is supported by many scientists [17].

Radioiodine treatment for differentiated thyroid cancer is associated with the application of, usually, high so called ablative activities of ^131^I (1.85 - 7.4 GBq), producing ionizing radiation at comparatively high level, resulting in significant radiation dose to the patient. Thus, unfavourable effects of such a treatment, in terms of oxidative damage to macromolecules, should be considered. It has been already documented, that oxidative damage to membrane lipids increases after more than 5 days [18]. However, the early effects of ^131^I in thyroid cancer patients on oxidative damage to macromolecules have never been examined.

Therefore, in the present study we examined the early effects of ablative activities of ^131^I in thyroid cancer patients on serum lipid peroxidation. The measurements were performed only within the first four days after the treatment, before levothyroxine replacement, to avoid possible favourable effects of such a replacement.

## Materials and methods

The study was carried out in 21 patients (18 females and 3 males, average age 52.4 ± 16.5 years) 4 weeks after total thyroidectomy for papillary (17 patients) and follicular (4 patients) thyroid carcinoma. After overnight fasting, blood samples were collected in the morning on the day of ^131^I administration before therapy (day "0") and daily thereafter until the 4^th ^day.

TSH concentration (BRAHMS, Germany) was measured in a sample obtained before therapy and concentrations of malondialdehyde + 4-hydroxyalkenals (MDA + 4-HDA), as the index of LPO, were measured in the blood serum by an LPO assay (LPO-586 kit, Calbiochem, USA) in all samples. Activity of 2.8 - 6.9 GBq of ^131^I was administered to the patients orally as sodium iodide (OBRI, Poland).

Sera from a group of 23 euthyroid patients (confirmed by normal thyroid hormone levels; fT3, fT4, TSH, all BRAHMS, Germany), matched for sex and age, served as controls.

### LPO assay

After collection, blood was centrifuged (3000 × g, 10 min, 4°C) in order to obtain serum, and stored at -80°C until assay. The concentrations of MDA and 4-HDA, as the index of LPO, were measured in blood serum, using an LPO-586 kit. Briefly, serum (200 ml) was mixed with 650 ml of a methanol:acetonitrile (1:3, v/v) solution, containing a chromogenic reagent, N-methyl-2-phenylindole, and vortexed. After adding 150 ml of methanesulfonic acid (15.4 M), incubation was carried out at 45°C for 40 min. The reaction between MDA + 4-HDA and N-methyl-2-phenylindole yields a chromophore, which is spectrophotometrically measured at the absorbance of 586 nm, using a solution of 4-hydroxynonenal (10 mM) as the standard. The level of LPO was expressed as the amount of MDA + 4-HDA (nmol) per 1 ml of serum.

### Statistical analysis

LPO in the study group on day "0" was compared with that of the control group, and LPO's in the study group on days 1-4 were compared with baseline value (Mann-Whitney U test and Wilcoxon's matched pairs test, respectively). Correlation between LPO and TSH, as well as between LPO and ^131^I was calculated (Spearman test).

The study protocol was accepted by the Ethical Committee of the Medical University of Lodz and written, informed consent was obtained from all patients.

## Results

Results are shown in Table 1.

**Table 1:** TSH concentration and LPO level (expressed as MDA + 4-HDA concentration) in the control group and hypothyroid patients treated with ^131^I.

	TSH (mIU/l)	MDA + 4-HDA (nmol/ml)				
		
		day "0"	day "1"	day "2"	day "3"	day "4"
Control patients	1.9 ± 0.5	4.9 ± 2.1				

Hypothyroid patients	60.2 ± 28.7	24.9 ± 12.7	22.2 ± 8.9	23.1 ± 10.0	23.9 ± 9.8	25.2 ± 8.0

All data represent mean ± SD

Expectedly, serum LPO level, when measured before ^131^I therapy, was several times higher (p < 0.00001) in cancer patients than in healthy subjects. We did not, however, observe any difference between LPO in hypothyroid patients after and before ^131^I therapy (day "1" *vs *day "0", p = 0.56; day "2" *vs *day "0", p = 0.83; day "3" *vs *day "0", p = 0.65; day "4" *vs *day "0", p = 0.80). No correlation was found between TSH and LPO on day "0" (r_s _= 0.22; p = 0.37). However, clear negative correlation was found between administered activity of ^131^I and LPO, when evaluated on day "2", being already at borderline level of statistical significance on day "1"; the negative correlation disappeared on day "3" and on day "4". Data are summarized in Table 2.

**Table 2:** Correlations between administered activity of ^131^I and LPO level in thyroid cancer patients. r_s _- correlation coefficient.

Day No	"1"	"2"	"3"	"4"
r_s _value	r_s _= -0.42	**r_s _= -0.57**	r_s _= -0.09	r_s _= -0.15

p value	p = 0.06	**p = 0.006**	p = 0.7	p = 0.54

Value being statistically significant is marked in bold.

## Discussion

Lipid peroxidation is a well-established indicator of oxidative stress in cells and tissues [19]. Lipid peroxides are unstable and decompose to form a series of compounds, including MDA and 4-HDA, and the measurement of MDA + 4-HDA has been used as an indicator of LPO [20,21].

In our study, we demonstrated increased LPO in the studied group of hypothyroid patients in comparison with the control group of patients with normal thyroid function. Our results are similar to those reported by Dumitriu *et al*., who found serum MDA level significantly higher in hypothyroidism when compared with the control group of euthyroid patients [10]. Furthermore, two independent groups described significant reduction of increased serum MDA levels after treatment of hypothyroidism with the use of levothyroxine [11,12]. Also Konukoğlu *et al*. found that increased LPO, when measured by thiobarbituric acid reactive substance (TBARS) method, was significantly reduced after treatment with levothyroxine [13]. The increased LPO level observed in both overt and subclinical hypothyroidism was recently explained by the insufficient increase in the antioxidant status, as well as by the altered lipid metabolism [22].

The main finding of the present study is that we did not demonstrate any additive effect of ablative doses of ^131^I on serum LPO in hypothyroid patients. Our results contrast with those reported by Konukoğlu *et al.*, who observed higher MDA levels in erythrocyte membranes 2 days after treatment with ^131^I in patients after subtotal thyroidectomy treated with 3.7 - 5.55 GBq ^131^I [23]. This apparent discordance may be explained by the high radiation sensitivity of bone marrow producing erythrocytes, the phenomenon which does relate to the present study. In turn, Wolfram *et al*. observed significant increase in isoprostane level in compartments such as blood plasma or serum and urine, shortly after radioiodine treatment in thyroid cancer patients [24]. As isoprostanes result from oxidation of low-density lipoprotein present in blood, whereas MDA + 4-HDA, evaluated in the present study, result from oxidative damage to membrane lipids of different tissues and organs, which thereafter are "released" into the blood, the values of these two parameters may change even divergently [24].

Also the following hypothesis to explain the apparent inconsistency between our results and those of other authors should be taken into consideration. It is known that total body irradiation after ^131^I administration rises with increasing whole-body retention time of radioisotope [25]. As no dosimetric data are available for patients studied by Konukoğlu *et al*., Bartoc *et al*., as well as Wolfram *et al*., it can be speculated that lower thyroid remnants ^131^I uptake and shorter whole-body retention time of ^131^I in our patients resulted in lower irradiation of tissues, and, as a consequence, lower LPO [18,23,24]. However, in the opinion of the authors of the present study, such a mechanism does not play an essential role.

The negative correlation between radioiodine dose and LPO level, observed in the present study early after radioiodine treatment was not expected. However, this relationship may be explained by well documented protective effects of ionizing radiation used in comparatively low doses [17,26]. Low doses of ionizing radiation, as well as low doses of any other prooxidant, stimulate intracellular protective mechanisms, antioxidative enzymes included [27]. It is worth stressing that in the present study LPO level decreased slightly within the first two days after radioiodine treatment and that this decrease would be possibly statistically significant in case of much lower baseline value (such as in the control group). Huge increase in LPO level due to severe hypothyroidism made impossible to clearly reveal the decrease in LPO level due to radioiodine treatment. However, this hypothesis should be experimentally proved, for example by measurement of other indices of oxidative damage to macromolecules. Nevertheless, the negative correlation between radioiodine dose and LPO level early after radioiodine treatment intensifies the main finding of the present study, showing that ablative doses of ^131^I do not induce significant peroxidation of membrane lipids in thyroid cancer patients within the first days of therapy. This finding is of great clinical importance and relates especially to those thyroid cancer patients, who are not treated with recombinant TSH, thus being hypothyroid not only before but also shortly after radioiodine therapy.

It is concluded that radioiodine remnant ablation of differentiated thyroid cancer does not further increase oxidative damage to membrane lipids, at least early, i.e. within the first 4 days, after therapy.

## Competing interests

The authors declare that they have no competing interests.

## Authors' contributions

JM participated in the design of the study, collected the material and participated in preparing the manuscript,

AL participated in the design of the study and participated in preparing the manuscript,

MK-L participated in the design of the study, carried out the assays and participated in preparing the manuscript.

All authors read and approved the final manuscript.
